# Three New Mitochondrial Genomes of Neritidae (Gastropoda, Neritimorpha) From China and Insights Into Their Phylogenetic Position

**DOI:** 10.1002/ece3.73888

**Published:** 2026-07-02

**Authors:** Yiyong Rao, Yuanzheng Meng, Sheng Zeng, Deyuan Yang

**Affiliations:** ^1^ South China Sea Fisheries Research Institute Chinese Academy of Fishery Sciences Guangzhou China; ^2^ College of the Environment and Ecology Xiamen University Xiamen China

**Keywords:** genome skimming, habitat transitions, mitochondrial genome, Neritidae, phylogeny, sequence curation

## Abstract

Marine‐to‐freshwater transitions are frequent in neritid snails. However, phylogenetic relationships among genera remain incompletely resolved, largely because many lineages lack mitogenome data and public records sometimes contain misidentified sequences. Here we assembled complete mitochondrial genomes for three Neritidae species from China—the seagrass specialist *Smaragdia rangiana*, the brackish‐water *Neripteron pileolus*, and the brackish‐to‐freshwater *Vittina cumingiana*—using genome skimming. The mitogenomes range from 15.68 to 15.82 kb, each containing the typical 37 genes and a major non‐coding region. All three mitogenomes are AT‐rich (62.8%–66.0%) with negative AT‐skews and positive GC‐skews. Codon usage analysis revealed that UCU (Serine) exhibits the highest relative synonymous codon usage across all three species, while nucleotide diversity among Neritidae PCGs is highest at third codon positions. Phylogenetic analyses were conducted using multiple datasets (13PCGs123, 13PCGs12, 13PCGsAA, with/without rRNA genes) under maximum likelihood and Bayesian inference, with TreeSpace analysis revealing three distinct topological clusters. All trees recovered the two‐subfamily framework (Neritinae vs. Neritininae). Within Neritininae, *S*. *rangiana* clustered with other *Smaragdia* sequences, *N*. *pileolus* grouped with *Neripteron violaceum*, and *V*. *cumingiana* formed a lineage sister to *Vitta* and *Puperita*. Several public sequences showed discordant placements consistent with historical synonymy or mislabeling, underscoring the need for voucher‐supported identifications. These new mitogenomes improve taxon sampling for Neritidae and provide a foundation for revisiting habitat transitions with broader genomic data.

## Graphical abstract

This study reports the newly assembled complete mitochondrial genomes for three neritid snails from China: *Smaragdia rangiana*, *Neripteron pileolus*, and *Vittina cumingiana*. Our phylogenetic analyses not only resolved their evolutionary positions within the established two‐subfamily framework but also identified several mislabeled public sequences, highlighting the critical need for voucher‐supported identifications in genomic databases.

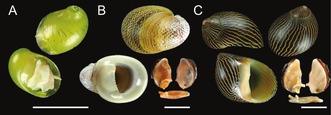

## 1 Introduction

Neritidae Rafinesque, 1815, commonly known as nerites, is a diverse family of gastropods with approximately 300 extant species (MolluscaBase eds. 2026). Neritids occupy a broad range of habitats, including tropical intertidal shores, mangroves, seagrass beds, and freshwater or brackish streams, and represent an important component of benthic macrofauna (Eichhorst 2016a; Lydeard and Cummings 2019). Habitat use is strongly partitioned within the family: roughly two‐thirds of neritid species are freshwater or brackish, whereas the remainder are marine. This ecological division is broadly reflected in taxonomy. Of the approximately 16 currently recognized genera, only three (*Nerita*, *Mienerita*, and *Smaragdia*) are strictly marine, while the remaining genera are primarily freshwater or brackish‐water dwellers. At the subfamily level, Neritidae is currently divided into two subfamilies, Neritinae Rafinesque, 1815 and Neritininae Poey, 1852. Neritinae contains the marine genera, *Nerita* and *Mienerita*, whereas Neritininae comprises the remaining genera, most of which inhabit freshwater or brackish environments (Eichhorst 2016a, 2016b; Bouchet et al. 2017; Lydeard and Cummings 2019).

Neritids show substantial variation in shell morphology, color patterns, life history, and habitat use. This diversity makes them useful model organisms for studying marine–freshwater transitions, adaptive radiation, and intertidal ecology, and also popular among collectors and aquarium hobbyists (Eichhorst 2016a; Ng et al. 2016). The repeated transitions between marine and freshwater environments have attracted particular attention. Holthuis (1995) proposed an evolutionary scenario for Neritidae based on life history and morphology, but this work predates modern taxonomic revisions and should be re‐evaluated within an explicit phylogenetic framework using molecular data.

The taxonomy of Neritidae has undergone repeated revisions at the genus and subfamily levels. Early classifications relied mainly on shell and operculum characters, whereas later systems incorporated radular and reproductive traits; habitat preference has also been used as supporting evidence (Eichhorst 2016a; Lydeard and Cummings 2019). For example, *Smaragdia* has sometimes been treated as a separate subfamily (Smaragdiinae) because of its distinctive seagrass‐associated ecology, and *Septaria* was previously elevated to family rank (Septariidae) due to its limpet‐like shell form (Wenz 1938; Golikov and Starobogatov 1975). Many freshwater neritids were historically placed in broad “catch‐all” genera such as *Theodoxus* or *Neritina*. Although molecular approaches have improved classification, many taxa still lack sequence data. The widely used modern system of Eichhorst (2016a) recognizes two subfamilies and 16 genera.

Phylogenetic analyses based on a few loci (e.g., cytochrome c oxidase subunit I, 16S rRNA, and ATP synthase subunit alpha) generally support the two‐subfamily framework (Frey and Vermeij 2008; Arquez et al. 2014). However, relationships among genera often remain weakly supported, and single or few markers may fail to capture processes such as introgression or incomplete lineage sorting. Complete mitochondrial genomes (mitogenomes) provide substantially more characters, typically comprising 13 protein‐coding genes, two ribosomal RNA genes, 22 transfer RNA genes, and a major non‐coding region, and have therefore become widely used in gastropod phylogenetics (Boore 1999). Recent neritid studies based on mitogenomes also recover the two‐subfamily split and improve resolution among some genera (Feng et al. 2021; Miao et al. 2022; Qi et al. 2023). Nevertheless, taxon coverage remains incomplete and public databases sometimes include outdated names or misidentified sequences, which can bias downstream phylogenetic inference if not carefully checked (Miao et al. 2022; Qi et al. 2023; Zeng et al. 2025; Lin et al. 2026).

Here, we report the complete mitochondrial genomes of three neritid species from China and assess their phylogenetic placements within Neritidae:

1. *Smaragdia rangiana* (Récluz, 1841), a widespread Indo‐Pacific marine species with an emerald‐green shell that lives exclusively on seagrasses. This is the first complete mitogenome reported for the genus *Smaragdia*.
2. *Neripteron pileolus* (Récluz, 1850), a brackish‐water species distributed from Japan to Malaysia that inhabits mangroves and mudflats at river mouths. Because it is frequently confused with *Neripteron violaceum* (Gmelin, 1791), we discuss potential misidentification issues in public records.
3. *Vittina cumingiana* (Récluz, 1842), a brackish‐to‐freshwater species from Southeast Asia commonly found in mangroves and swamps and widely traded in the aquarium hobby. This is the first complete mitogenome reported for the genus *Vittina*.

## 2 Materials and Methods

### 2.1 Specimen Collection, Identification and Sequencing

**FIGURE 1:**
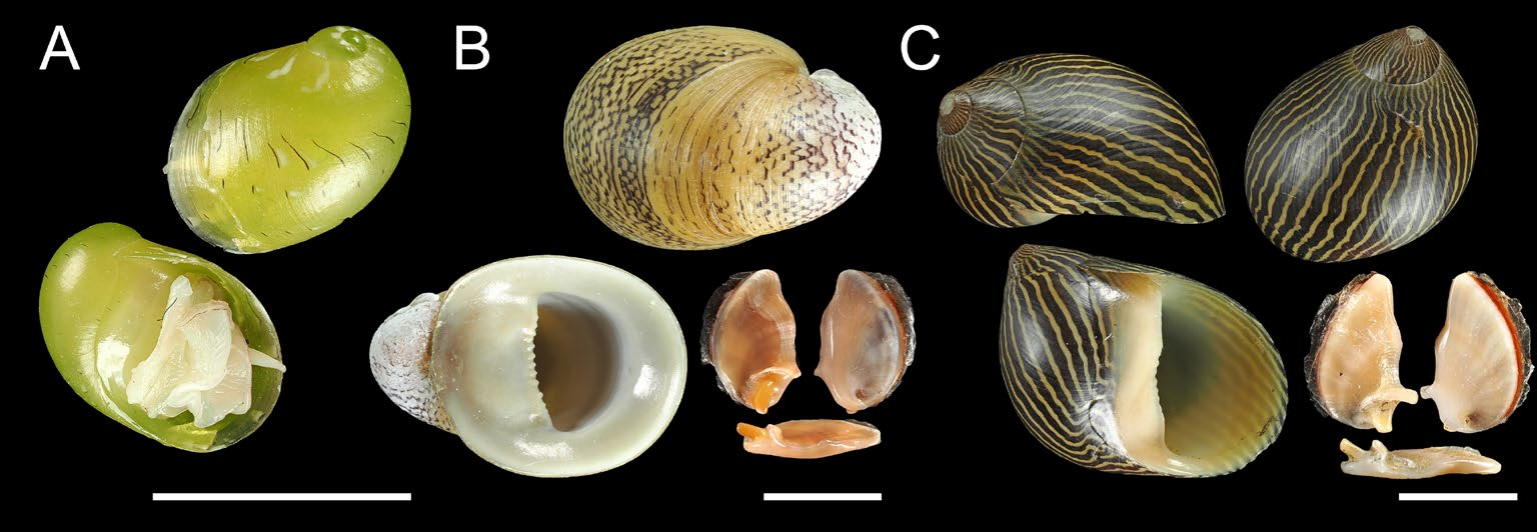
Specimens used in this study. (A) *Smaragdia rangiana* (Récluz, 1841), NER‐004; (B) *Neripteron pileolus* (Récluz, 1850), NER‐037; (C) *Vittina cumingiana* (Récluz, 1842), NER‐038. The scale bar = 5 mm.

Specimens were collected from Shanghai and Hainan, China (Figure 1 and Table 1) and identified morphologically following (Eichhorst 2016a, 2016b). No specific permits were required for the collection of these gastropod specimens under Chinese law, as the sampling sites are not located in protected areas and the species collected are neither endangered nor regulated. All collections were conducted in compliance with local regulations. To further confirm taxonomic identity, we conducted BLAST searches against NCBI using assembled mitogenome sequences and reconstructed phylogenetic trees incorporating all available cytochrome c oxidase subunit I and 16S rRNA sequences of Neritidae from NCBI. The detailed BLAST workflow follows Yang et al. (2024). Specimens were fixed in ≥ 99.7% ethanol, stored at −20°C, and deposited at the College of the Environment and Ecology, Xiamen University (voucher numbers: see Table 1).

**TABLE 1:** Sample information for the three neritid specimens sequenced in this study.

Scientific name	Specimen catalog	Collection date	Locality	Latitude	Longitude	Accession number
*Smaragdia rangiana* (Récluz, 1841)	NER‐004	2024–06	On the seagrass, in a lagoon of Lingshui, Hainan Province, China	18°25′0″ N	110°2′48″ E	PX869896
*Neripteron pileolus* (Récluz, 1850)	NER‐037	2024–10	On rocks of the intertidal zone, Nanhui District, Shanghai, China	30°54′24″ N	121°58′24″ E	PX869897
*Vittina cumingiana* (Récluz, 1842)	NER‐038	2024–04	Lingshui River, Lingshui, Hainan Province, China	18°30′1″ N	110°4′51″ E	PX869898

Before DNA extraction, specimens were rinsed with ≥ 97% ethanol to minimize potential surface contamination. Foot tissue was dissected to reduce potential gut‐derived contamination. Whole genomic DNA was extracted using the TIANamp Genomic DNA Kit (TIANGEN, Beijing, China). Genome skimming was conducted by Novogene Bioinformatics Technology Co. Ltd. (Beijing, China), using paired‐end 150 bp sequencing on an Illumina NovaSeq X Plus platform. The generated raw sequencing data for each specimen were 3.86 GB (NER‐004), 3.91 GB (NER‐037), and 4.44 GB (NER‐038).

### 2.2 Assembly and Annotation of Mitochondrial Genome

Raw paired‐end reads were quality‐filtered using fastp v0.23.4 (Chen et al. 2018) to remove adapter sequences and trim low‐quality bases. Mitogenomes were assembled using GetOrganelle v1.7.6.1 (Jin et al. 2020) with multiple k‐mer sizes (17, 21, 33, 39, 45, 55, 65, 75, 85, 95, 105, 115, and 127); all other parameters followed Xu et al. (2024); Yang et al. (2024).

Mitogenomes were annotated using MitoFinder v1.4.1 (Allio et al. 2020) and MitoZ v3.6 (Meng et al. 2019). GenBank‐format annotation files (.gb) were reorganized by custom python script using COX1 as the first gene (Yang et al. 2024). Annotations were then manually curated in Geneious Prime v2022.2.2 (Kearse et al. 2012) following the criteria of Yang et al. (2024).

### 2.3 Sequence Analyses

Strand asymmetries were calculated using the formulas proposed by Perna and Kocher (1995): AT‐skew = (A − T)/(A + T) and GC‐skew = (G − C)/(G + C). Codon usage and relative synonymous codon usage (RSCU) of the 13 protein‐coding genes (PCGs) were analyzed using PhyloSuite and visualized with the ‘ggplot2’ package (Wickham and Wickham 2016) in R v4.1.3 (R Core Team 2023). Non‐synonymous (Ka) to synonymous (Ks) substitution rate ratios and Nucleotide diversity (Pi) were estimated using DnaSP v.6.0 (Rozas et al. 2017) for all Neritidae sequences listed in Table 2.

**TABLE 2:** List of 54 sequences and three outgroups used for phylogenetic analysis.

ID	Organism	Family	Length	AT%	For TreeSpace
MW694825.1	*Clithon corona*	Neritidae	15,975	64.8	
NC_062611.1	*Clithon corona*	Neritidae	15,975	64.8	1
MW694826.1	*Clithon lentiginosum*	Neritidae	15,885	64.8	
NC_062610.1	*Clithon lentiginosum*	Neritidae	15,885	64.8	1
MT568501.1	*Clithon oualaniense*	Neritidae	15,706	65.8	
NC_068076.1	*Clithon oualaniense*	Neritidae	15,712	65.7	1
KU237287.1	*Clithon retropictum*	Neritidae	15,803	64.9	
MG190355.1	*Clithon retropictum*	Neritidae	15,802	64.9	
NC_031893.1	*Clithon retropictum*	Neritidae	15,814	64.9	1
MT230542.1	*Clithon sowerbianum*	Neritidae	15,919	64.5	
NC_068077.1	*Clithon sowerbianum*	Neritidae	15,895	64.4	1
MW694827.1	*Clithon squarrosum*	Neritidae	15,905	65.0	
NC_062613.1	*Clithon squarrosum*	Neritidae	15,905	65.0	1
NC_060871.1	*Neritina violacea*	Neritidae	15,618	65.8	1
MK516738.1	*Nerita albicilla*	Neritidae	15,314	64.5	1
PQ285859.1	*Nerita albicilla*	Neritidae	15,634	64.7	
MN477253.1	*Nerita balteata*	Neritidae	15,571	63.3	1
MT161611.1	*Nerita chamaeleon*	Neritidae	15,716	65.8	1
NC_068078.1	*Nerita costata*	Neritidae	15,604	61.6	1
KF728888.1	*Nerita fulgurans*	Neritidae	15,343	64.4	1
NC_068083.1	*Nerita histrio*	Neritidae	15,538	65.5	1
NC_068079.1	*Nerita insculpta*	Neritidae	15,721	61.5	1
MN747116.1	*Nerita japonica*	Neritidae	15,875	65.2	1
LC565707.1	*Nerita japonica*	Neritidae	15,877	65.2	
GU810158.1	*Nerita melanotragus*	Neritidae	15,261	63.5	1
NC_068080.1	*Nerita ocellata*	Neritidae	15,577	63.8	1
PQ310099.1	*Nerita originalis*	Neritidae	15,746	64.4	1
OZ373285.1	*Nerita plicata*	Neritidae	16,689	63.5	
NC_068081.1	*Nerita plicata*	Neritidae	15,737	61.6	1
NC_068082.1	*Nerita reticulata*	Neritidae	15,630	65.3	1
KF728889.1	*Nerita tessellata*	Neritidae	15,741	64.0	1
MN477254.1	*Nerita undata*	Neritidae	15,583	63.2	
NC_087859.1	*Nerita undata*	Neritidae	15,878	62.8	1
KF728890.1	*Nerita versicolor*	Neritidae	15,866	61.6	1
MK395169.1	*Nerita yoldii*	Neritidae	15,719	64.7	1
MW694828.1	*Neritina iris*	Neritidae	15,618	64.3	
NC_062612.1	*Neritina iris*	Neritidae	15,618	64.3	1
KY021066.1	*Neritina violacea*	Neritidae	15,710	66.3	1
PP958837.1	*Neritona juttingae*	Neritidae	15,950	62.7	1
MZ315041.1	*Septaria lineata*	Neritidae	15,697	65.8	
NC_064130.1	*Septaria lineata*	Neritidae	15,697	65.8	1
KU342667.1	*Theodoxus fluviatilis*	Neritidae	14,122	67.6	
MT628587.1	*Theodoxus fluviatilis*	Neritidae	15,667	66.1	1
PX869896	*Smaragdia rangiana*	Neritidae	15,824	62.8	1
PX869897	*Neripteron pileolus*	Neritidae	15,794	66.0	1
PX869898	*Vittina cumingiana*	Neritidae	15,681	63.0	1
KU342665.1	*Vitta usnea*	Neritidae	15,574	64.0	1
SRR1505122	*Nerita peloronta*	Neritidae	—	—	
SRR8318357	*“Clithon parvulum”*	Neritidae	—	—	
SRR19577567	*Smaragdia pulcherrima*	Neritidae	—	—	
SRR8318353	*Smaragdia rangiana*	Neritidae	—	—	
SRR11015441	*Neritina pulligera*	Neritidae	—	—	
SRR8318346	*Neritina* sp.	Neritidae	—	—	
SRR8318350	*Puperita pupa*	Neritidae	—	—	
KU342664.1	*Georissa bangueyensis*	Hydrocenidae	15,267	68.8	1
KU342666.1	*Pleuropoma jana*	Helicinidae	15,851	74.6	1
KU342669.1	*Titiscania limacina*	Neritopsidae	15,046	61.8	1

### 2.4 Phylogenetic Analysis

We downloaded all publicly available Neritidae mitogenome records from the NCBI Nucleotide/GenBank database (62 accessions available as of 1 January 2026). Because redundancy can occur in GenBank (e.g., identical or near‐identical mitogenome sequences deposited under different accession numbers), we dereplicated the dataset in PhyloSuite and retained unique sequences. After filtering, 44 non‐redundant mitogenomes were kept for downstream analyses. To ensure consistent annotation criteria and to correct potential errors in database records, we re‐annotated all mitogenomes using the workflow described above (see also Yang et al. 2024).

To increase taxon sampling, we additionally retrieved publicly available Neritidae transcriptome datasets from the NCBI Sequence Read Archive (SRA) and extracted mitochondrial genes following the pipeline described by Zeng et al. (2025). Briefly, the target mitochondrial genes were annotated and extracted from the transcriptomic data using MitoFinder v1.4 (Allio et al. 2020). Complete mitochondrial genomes were not obtained from these datasets; instead, we successfully assembled and retrieved all 13 protein‐coding genes (PCGs) and the two ribosomal RNAs (12S and 16S). Our preliminary phylogenetic analyses based on the 13PCGs + 2rRNA dataset demonstrated that sequences belonging to the same species formed monophyletic groups (Data S2). Therefore, to minimize sequence redundancy and streamline the phylogenetic dataset, we selected a single representative sequence for each species for the final analyses.

Ultimately, we analyzed two datasets: (1) a combined dataset including mitogenomes and transcriptome‐derived mitochondrial genes (“mitogenomes + RNA”); and (2) a mitogenome‐only dataset (“mitogenomes”).

For the mitogenome‐only dataset, we constructed several alignments to evaluate sensitivity to coding position and partitioning: (i) 13PCGs123, all codon positions of the 13 protein‐coding genes; (ii) 13PCGs123 + 2rRNA, 13PCGs123 plus the two rRNA genes; (iii) 13PCGs12, first and second codon positions of the 13 protein‐coding genes; (iv) 13PCGs12 + 2rRNA, 13PCGs12 plus the two rRNA genes; (v) 13PCGsAA, amino acid translations of the 13 protein‐coding genes; (vi) 13PCGs‐np, 13 protein‐coding genes without codon partitioning; and (vii) 13PCGs + 2rRNA‐np, 13 protein‐coding genes plus the two rRNA genes without partitioning. Three Cycloneritida taxa, *Titiscania limacina* (KU342669, Neritopsidae), *Georissa bangueyensis* (KU342664, Hydrocenidae), and *Pleuropoma jana* (KU342666, Helicinidae) were used as outgroups (Qi et al. 2023).

TrimAl v1.2 (Capella‐Gutiérrez et al. 2009) was employed to remove ambiguously aligned regions using the ‘automated1’ setting (Data S1 and Table S1). Substitution models were selected using ModelFinder v2.2.0 (Kalyaanamoorthy et al. 2017) based on the Bayesian Information Criterion (BIC) for maximum likelihood (ML) analysis and the corrected Akaike Information Criterion (AICc) for Bayesian inference (BI) analysis under a partitioned model (Data S1 and Table S2). ML phylogenies were performed using IQ‐TREE v2.2.2 (Nguyen et al. 2015; Kalyaanamoorthy et al. 2017) with the best partition scheme and an edge‐linked partition model. Node support was evaluated with 100,000 ultrafast bootstraps. BI phylogenies were constructed in MrBayes v3.2.7a (Ronquist et al. 2012), with two parallel runs and 2000, 000 generations. Convergence was assessed by the average standard deviation of split frequencies (ASDSF), with additional generations added if the ASDSF value exceeded 0.005.

Topological discrepancies between the resulting trees were evaluated using TreeSpace (Jombart et al. 2017) in R v4.3.1 (R Core Team 2023), which employs Metric Multidimensional Scaling (MDS) to visualize tree relationships and hierarchical clustering methods (including single linkage, complete linkage, UPGMA, and Ward's method) to formally identify distinct phylogenetic clusters. Finally, phylogenetic trees were visualized and edited using iTOL v6 (Letunic and Bork 2024).

## 3 Results

### 3.1 Species Identification

Species identity was assessed using both morphology and mitochondrial markers (cytochrome c oxidase subunit I and 16S rRNA; Data S1 and Table S3). Live individuals were identified morphologically following Eichhorst (2016a, 2016b) (Figure 1) and further validated by BLAST searches and marker‐based phylogenies.

*Smaragdia rangiana* (Récluz, 1841), NER‐004 (Figure 1A): Shell small, thin, elongated semi‐globose. Surface smooth. Spire prominent. Three whorls, and the body whorl is greatly expanded. Parietal shelf convex and very swollen, cream color with green tints. Columella fairly straight with five to six teeth. Shell yellowish‐green (bright green when alive), with up to five lines of white blotches or bars, and each white blotch has a black line on the leading edge. Distinguished by distribution (Indo‐Pacific) from *S. viridis* (tropical Atlantic). Molecular validation via phylogenetic analysis, using mitochondrial genome sequences assembled from publicly available transcriptome data (SRR8318353), supported the morphological identification (Data S1 and Table S3).

*Neripteron pileolus* (Récluz, 1850), NER‐037 (Figure 1B): Shell medium size, solid, elongated ovate. Final whorl flared into an oval base with a sharp lip that extends completely 360° around the base. Spire curved in on itself, extended beyond the parietal shelf. Surface always eroded, with apparent axial growth lines. Parietal shelf slightly concave, smooth. Aperture and parietal shelf cream color with bluish‐gray staining. A long, shallow indentation on the columella, with 10–25 small teeth. Shell greenish or yellowish brown, irregular zigzag lines forming tent or flame patterns. Distinguished by a bluish‐gray parietal shelf from *N. violacea* (red) and *N. cornucopia* (black). Molecular validation showed high identity (over 99% of COI, 16S, and mtgenome) to *Neritina violacea* sequences, but Lin et al. (2026) mentioned that some records labeled as *N. violacea* are actually *Neripteron pileolus* (Data S1 and Table S3).

*Vittina cumingiana* (Récluz, 1842), NER‐038 (Figure 1C): Shell medium size, elongated ovate. Surface glossy and smooth. Spire high and prominent. Apex eroded, with about three whorls left. Parietal shelf convex, swollen, with leather‐like microscopic texture; cream to peach or orange color. A central depression on the columella with 7–10 teeth. Shell black, with a series of thin, parallel, zigzag, yellow spiral stripes. Distinguished by narrow spiral stripes from *V. turrita* (with broad spiral stripes) and by a high spire from other similar congeners (with moderate spires). Molecular validation supported assignment to the genus *Vittina* (Data S1 and Table S3).

### 3.2 Mitogenome Organization

**FIGURE 2:**
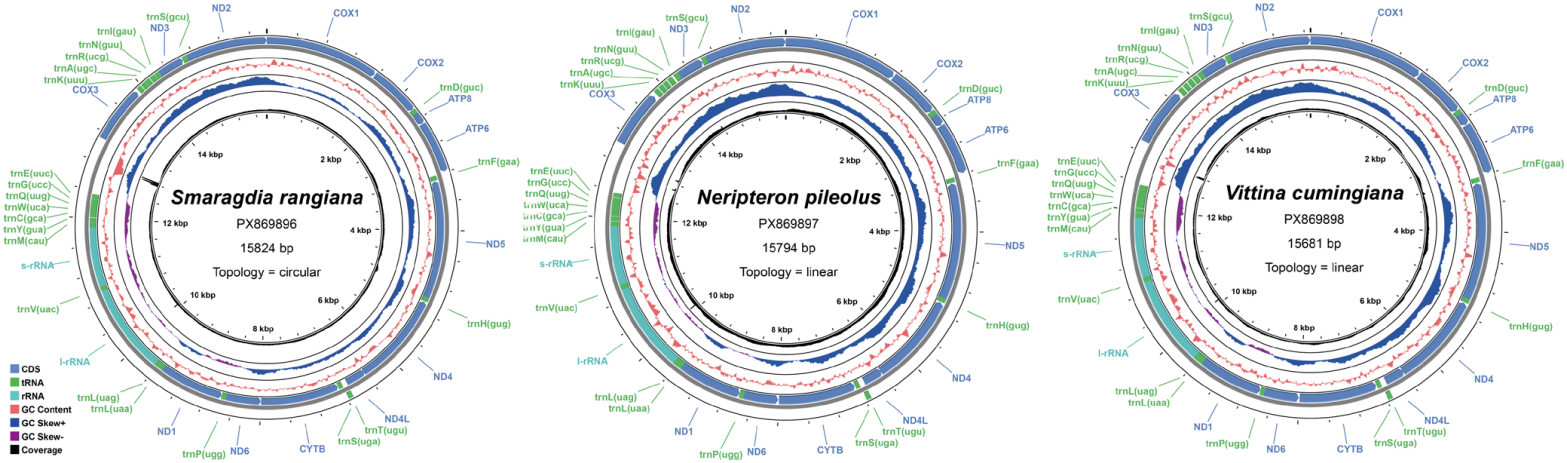
Gene maps of the *Smaragdia rangiana* (NER‐004), *Neripteron pileolus* (NER‐037), and *Vittina cumingiana* (NER‐038) mitogenomes. The innermost and middle circles depict the distribution of the sequencing depth, GC content, and GC‐skew, respectively. The outermost circle represents the arrangement of genes: outer genes from the forward strand, and inner genes from the reverse strand.

We assembled complete mitogenomes for three species: *Smaragdia rangiana* (15,824 bp; NER‐004; PX869896), *Neripteron pileolus* (15,794 bp; NER‐037; PX869897), and *Vittina cumingiana* (15,681 bp; NER‐038; PX869898) (Figure 2). Consistent with previously reported Neritidae mitogenomes, the structural characteristics of these three newly sequenced genomes are strictly conserved. Each mitogenome contained the typical set of 37 genes (including 13 protein‐coding genes and two rRNAs) and a major non‐coding region (Table 3), exhibiting no gene rearrangements (Lin et al. 2026). The overall A + T content was 62.8% in *S. rangiana*, 66.0% in *N*. *pileolus*, and 63.0% in *V*. *cumingiana* (Table 4), falling within the range reported for other neritid mitogenomes (Table 2).

**TABLE 3:** Features of three species' mitogenomes (*S. rangiana*/*N. pileolus*/*V. cumingiana*).

Genes	Position		Size	Intergenic nucleotides	Codon		Strand
	From	To			Start	Stop	
COX1	1/1/1	1548/1548/1548	1548/1548/1548	11/11/11	ATG/ATG/ATG	TAA/TAA/TAA	H/H/H
COX2	1560/1560/1560	2249/2249/2249	690/690/690	1/1/1	ATG/ATG/ATG	TAA/TAG/TAA	H/H/H
trnD(guc)	2251/2251/2251	2316/2316/2316	66/66/66	0/0/0			H/H/H
ATP8	2317/2317/2317	2481/2481/2481	165/165/165	5/6/6	ATG/ATG/ATG	TAA/TAA/TAA	H/H/H
ATP6	2487/2488/2488	3188/3189/3189	702/702/702	22/22/22	ATG/ATG/ATG	TAA/TAA/TAA	H/H/H
trnF(gaa)	3211/3212/3212	3278/3279/3279	68/68/68	9/9/9			L/L/L
ND5	3288/3289/3289	4992/4993/4993	1705/1705/1705	0/0/0	ATG/ATG/ATG	T/T/T	L/L/L
trnH(gug)	4993/4994/4994	5058/5059/5059	66/66/66	9/9/9			L/L/L
ND4	5068/5069/5069	6424/6425/6425	1357/1357/1357	−7/−7/−7	ATG/ATG/ATG	T/T/T	L/L/L
ND4L	6418/6419/6419	6711/6712/6712	294/294/294	4/4/4	ATG/ATG/ATG	TAA/TAA/TAA	L/L/L
trnT(ugu)	6716/6717/6717	6783/6784/6784	68/68/68	3/5/6			H/H/H
trnS(uga)	6787/6790/6791	6851/6854/6855	65/65/65	5/5/5			L/L/L
CYTB	6857/6860/6861	7993/7996/7997	1137/1137/1137	5/10/11	ATG/ATG/ATG	TAA/TAA/TAA	L/L/L
ND6	7999/8007/8009	8505/8513/8515	507/507/507	1/1/1	ATG/ATG/ATG	TAA/TAA/TAA	L/L/L
trnP(ugg)	8507/8515/8517	8572/8580/8582	66/66/66	1/0/1			L/L/L
ND1	8574/8581/8584	9506/9513/9516	933/933/933	0/0/0	ATG/ATG/ATG	TAG/TAA/TAA	L/L/L
trnL(uaa)	9507/9514/9517	9574/9581/9584	68/68/68	0/0/0			L/L/L
trnL(uag)	9575/9582/9585	9644/9650/9654	70/69/70	0/0/0			L/L/L
l‐rRNA	9645/9651/9655	10,943/10949/10954	1299/1299/1300	0/0/0			L/L/L
trnV(uac)	10,944/10950/10955	11,010/11017/11022	67/68/68	0/0/0			L/L/L
s‐rRNA	11,011/11018/11023	11,867/11879/11886	857/862/864	0/0/0			L/L/L
trnM(cau)	11,868/11880/11887	11,934/11946/11953	67/67/67	2/5/4			L/L/L
trnY(gua)	11,937/11952/11958	12,004/12019/12025	68/68/68	7/5/5			L/L/L
trnC(gca)	12,012/12025/12031	12,075/12089/12094	64/65/64	0/0/0			L/L/L
trnW(uca)	12,076/12090/12095	12,142/12155/12160	67/66/66	0/0/0			L/L/L
trnQ(uug)	12,143/12156/12161	12,211/12224/12229	69/69/69	0/0/0			L/L/L
trnG(ucc)	12,212/12225/12230	12,276/12289/12295	65/65/66	2/6/1			L/L/L
trnE(uuc)	12,279/12296/12297	12,344/12361/12362	66/66/66	773/683/580			L/L/L
COX3	13,118/13045/12943	13,897/13824/13722	780/780/780	28/39/31	ATG/ATG/ATG	TAA/TAA/TAA	H/H/H
trnK(uuu)	13,926/13864/13754	13,991/13930/13820	66/67/67	10/11/16			H/H/H
trnA(ugc)	14,002/13942/13837	14,069/14009/13904	68/68/68	3/8/14			H/H/H
trnR(ucg)	14,073/14018/13919	14,141/14086/13987	69/69/69	9/14/12			H/H/H
trnN(guu)	14,151/14101/14000	14,223/14172/14071	73/72/72	4/26/12			H/H/H
trnI(gau)	14,228/14199/14084	14,296/14267/14152	69/69/69	0/0/0			H/H/H
ND3	14,297/14268/14153	14,650/14621/14506	354/354/354	4/3/5	ATG/ATG/ATG	TAA/TAA/TAG	H/H/H
trnS(gcu)	14,655/14625/14512	14,721/14692/14579	67/68/68	1/0/0			H/H/H
ND2	14,723/14693/14580	15,824/15794/15681	1102/1102/1102	0/0/0	ATG/ATG/ATG	T/T/T	H/H/H

**TABLE 4:** Composition and skewness of three species' mitogenomes (*S. rangiana*/ *N. pileolus*/ *V. cumingiana*).

Regions	Size (bp)	T(U)	C	A	G	AT (%)	GC (%)	GT (%)	AT skewness	GC skewness
Full genome	15,824/15794/15681	32.2/34.6/32.8	16.7/14.4/16	30.6/31.4/30.2	20.5/19.6/21	62.8/66/63	37.2/34/37	52.7/54.2/53.8	−0.026/−0.049/−0.04	0.101/0.152/0.135
PCGs	11,271/11271/11271	37.3/38.7/37.2	19.1/17.2/19	24.1/26.7/24.9	19.5/17.5/19	61.4/65.4/62.1	38.6/34.7/38	56.8/56.2/56.2	−0.214/−0.184/−0.198	0.011/0.009/0.002
tRNAs	1482/1483/1484	31.6/32.6/32.4	15.4/14.9/15.1	30.7/30.5/30.4	22.3/21.9/22.1	62.3/63.1/62.8	37.7/36.8/37.2	53.9/54.5/54.5	−0.015/−0.033/−0.032	0.183/0.19/0.188
rRNAs	2156/2161/2164	29.4/32.2/30.9	16.8/15.5/16.4	35.6/35.5/34.8	18.2/16.8/17.9	65/67.7/65.7	35/32.3/34.3	47.6/49/48.8	0.096/0.049/0.059	0.04/0.043/0.043
1st codon position	3757/3757/3757	29.8/30.7/30.3	17.9/16.9/17.5	25.6/26.1/25.5	26.7/26.2/26.7	55.4/56.8/55.8	44.6/43.1/44.2	56.5/56.9/57	−0.077/−0.082/−0.086	0.196/0.216/0.208
2nd codon position	3757/3757/3757	43/43/43.3	22.7/22.4/22.2	18/18.2/18	16.4/16.5/16.5	61/61.2/61.3	39.1/38.9/38.7	59.4/59.5/59.8	−0.411/−0.406/−0.413	−0.161/−0.151/−0.146
3rd codon position	3757/3757/3757	39.1/42.3/37.9	16.6/12.2/17.2	28.9/35.7/31.1	15.4/9.7/13.8	68/78/69	32/21.9/31	54.5/52/51.7	−0.15/−0.084/−0.098	−0.039/−0.114/−0.107
ATP6	702/702/702	39.5/43.3/39.9	17.2/14.7/18.2	22.1/22.4/21.5	21.2/19.7/20.4	61.6/65.7/61.4	38.4/34.4/38.6	60.7/63/60.3	−0.282/−0.319/−0.299	0.104/0.145/0.055
ATP8	165/165/165	39.4/41.2/41.2	12.7/10.3/10.9	28.5/30.9/30.3	19.4/17.6/17.6	67.9/72.1/71.5	32.1/27.9/28.5	58.8/58.8/58.8	−0.161/−0.143/−0.153	0.208/0.261/0.234
COX1	1548/1548/1548	37.6/40.4/38.8	18.1/15.3/16	22.9/23/23	21.4/21.3/22.2	60.5/63.4/61.8	39.5/36.6/38.2	59/61.7/61	−0.242/−0.275/−0.256	0.083/0.163/0.161
COX2	690/690/690	35.4/36.4/35.5	17.4/14.5/16.1	26.4/28.4/27.7	20.9/20.7/20.7	61.8/64.8/63.2	38.3/35.2/36.8	56.3/57.1/56.2	−0.146/−0.123/−0.124	0.091/0.177/0.126
COX3	780/780/780	36.9/38.8/38.1	18.7/16.2/17.1	20.6/22.4/21.4	23.7/22.6/23.5	57.5/61.2/59.5	42.4/38.8/40.6	60.6/61.4/61.6	−0.283/−0.268/−0.28	0.118/0.166/0.158
CYTB	1137/1137/1137	35.4/36.7/36.1	22/20.3/22.1	24.9/27.8/25.1	17.8/15.2/16.7	60.3/64.5/61.2	39.8/35.5/38.8	53.2/51.9/52.8	−0.174/−0.138/−0.181	−0.106/−0.144/−0.138
ND1	933/933/933	36.5/37.2/37	20.5/19.2/19.2	23.5/27/25.4	19.5/16.6/18.4	60/64.2/62.4	40/35.8/37.6	56/53.8/55.4	−0.218/−0.159/−0.186	−0.024/−0.072/−0.02
ND2	1102/1102/1102	38.9/43.3/39.8	13.8/10.6/13.2	22.6/24.2/22.1	24.7/21.9/24.9	61.5/67.5/61.9	38.5/32.5/38.1	63.6/65.2/64.7	−0.265/−0.282/−0.286	0.283/0.346/0.308
ND3	354/354/354	40.1/44.4/41.5	13.6/10.7/13	20.1/19.8/20.3	26.3/25.1/25.1	60.2/64.2/61.8	39.9/35.8/38.1	66.4/69.5/66.6	−0.333/−0.383/−0.342	0.319/0.402/0.319
ND4	1357/1357/1357	38.7/37.2/35.5	20.9/21.4/23.7	24.8/29.3/26.9	15.5/12.1/13.9	63.5/66.5/62.4	36.4/33.5/37.6	54.2/49.3/49.4	−0.218/−0.118/−0.138	−0.147/−0.278/−0.263
ND4L	294/294/294	34.7/34.4/31.3	19.4/18.4/21.8	28.9/30.3/29.3	17/17/17.7	63.6/64.7/60.6	36.4/35.4/39.5	51.7/51.4/49	−0.091/−0.063/−0.034	−0.065/−0.038/−0.103
ND5	1705/1705/1705	35.9/35.4/34.8	22.7/20.9/22.8	26.1/31/27.3	15.3/12.7/15	62/66.4/62.1	38/33.6/37.8	51.2/48.1/49.8	−0.158/−0.067/−0.121	−0.194/−0.243/−0.206
ND6	507/507/507	39.4/40/37.5	18.1/17.2/20.1	26/29.6/26	16.4/13.2/16.4	65.4/69.6/63.5	34.5/30.4/36.5	55.8/53.2/53.9	−0.205/−0.15/−0.18	−0.051/−0.13/−0.103
l‐rRNA	1299/1299/1300	29.2/33.3/32	17.2/15.5/16.4	36.4/35.6/34.8	17.2/15.6/16.8	65.6/68.9/66.8	34.4/31.1/33.2	46.4/48.9/48.8	0.11/0.033/0.043	−0.002/0.002/0.012
s‐rRNA	857/862/864	29.6/30.5/29.3	16.2/15.4/16.4	34.3/35.3/34.7	19.8/18.8/19.6	63.9/65.8/64	36/34.2/36	49.4/49.3/48.9	0.073/0.072/0.085	0.1/0.098/0.087

When calculated from the coding strand of individual genes, the A + T content of the 13 protein‐coding genes was 61.4% (*S*. *rangiana*), 65.4% (*N*. *pileolus*), and 62.1% (*V*. *cumingiana*) (Table 4). Among the protein‐coding genes (PCGs), ATP8 exhibited the highest A + T content, ranging from 67.9% to 72.1% across the three species (*S*. *rangiana*: 67.9%; *N*. *pileolus*: 72.1%; *V*. *cumingiana*: 71.5%), while COX3 displayed the lowest, varying from 57.5% to 61.2% (*S. rangiana*: 57.5%; *N. pileolus*: 61.2%; *V*. *cumingiana*: 59.5%) (Table 3).

AT‐skew values were negative across all three species, whereas GC‐skew values were positive. AT‐skew values followed the pattern: −0.026 (*S*. *rangiana*) > −0.040 (*V*. *cumingiana*) > −0.049 (*N*. *pileolus*). GC‐skew values demonstrated the order: 0.152 (*N*. *pileolus*) > 0.135 (*V*. *cumingiana*) > 0.101 (*S*. *rangiana*) (Table 4).

### 3.3 Genes and Codon Usage

Among the 13 protein‐coding genes, all utilized the standard initiation codon ATG (Table 3). All 13 genes terminated with canonical stop codons (TAG, TAA, and T). Transfer RNA gene lengths varied from 64 to 73 bp. The 16S rRNA gene length was 1299 bp (*S*. *rangiana* and *V*. *cumingiana*) and 1300 bp (*N*. *pileolus*), while the 12S rRNA gene was 857 bp (*S*. *rangiana*), 862 bp (*V*. *cumingiana*), and 864 bp (*N*. *pileolus*) (Table 3). The non‐coding region (NCR), located between trnE and COX3, was 780 bp in three mitogenomes.

**FIGURE 3:**
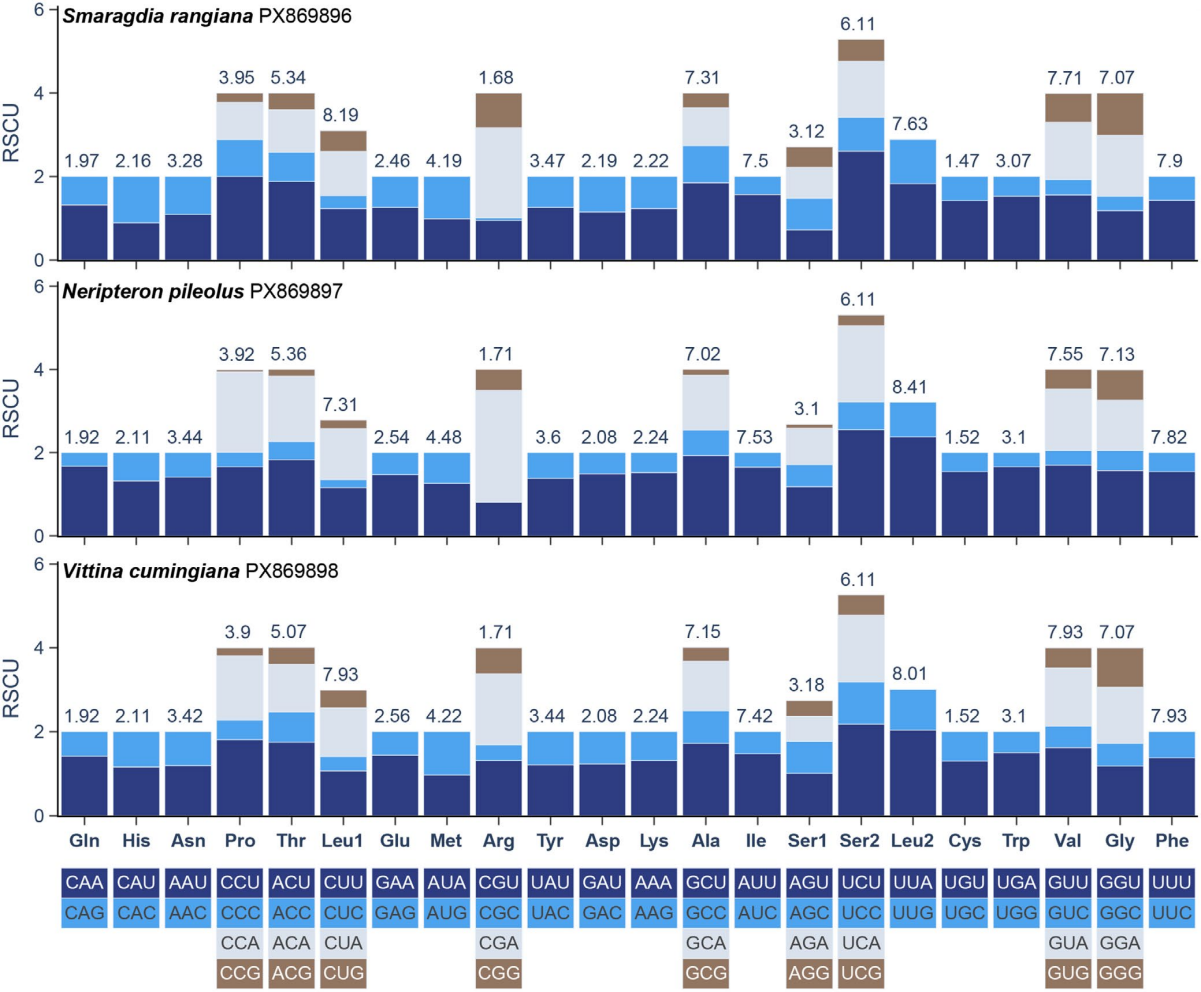
Relative synonymous codon usage (RSCU) of the three mitogenomes. The codon families are provided under the x‐axis. The frequency of amino acid usage is listed above the column.

Relative synonymous codon usage (RSCU) analysis revealed that UCU (S) exhibited the highest RSCU values across all three species: 2.61 (*S*. *rangiana*), 2.55 (*V*. *cumingiana*), and 2.18 (*N*. *pileolus*). The lowest RSCU values were observed for CCG (P) in *S*. *rangiana* (0.22), *V*. *cumingiana* (0.05), and *N*. *pileolus* (0.19) (Figure 3, Data S1 and Table S4).

### 3.4 Nucleotide Diversity and Evolutionary Rate Analysis

**FIGURE 4:**
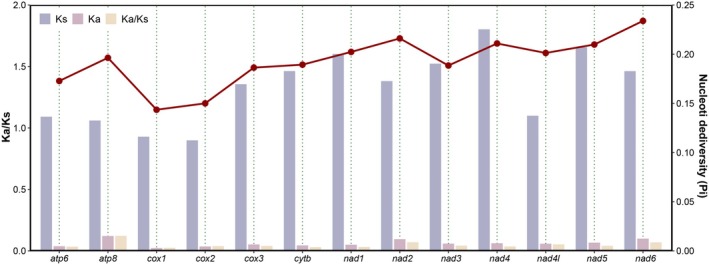
Nucleotide diversity and Ka/Ks rates of 13 PCGs based on Neritidae sequences. The light purple, pink, and light yellow columns represent the values of Ks, Ka, and Ka/Ks, respectively. The red dot represents the value of nucleotide diversity (Pi).

Nucleotide diversity (Pi) of the analyzed genes ranged from 0.144 to 0.234. Sequence variability was lowest in COX1 (Pi = 0.144), COX2 (0.150), ATP6 (0.173), and COX3 (0.186), and highest in ND6 (0.234), ND2 (0.216), and ND4 (0.211). Non‐synonymous to synonymous substitution ratio (Ka/Ks) analysis revealed that all genes are under purifying selection (Ka/Ks < 1). Evolutionary rates were comparatively slow for COX1 (Ka/Ks = 0.024), CYTB (0.030), ND1 (0.032), and ATP6 (0.034), but relatively fast for ATP8 (0.122), ND2 (0.070), and ND6 (0.069) (Figure 4).

### 3.5 Phylogenetic Analysis

**FIGURE 5:**
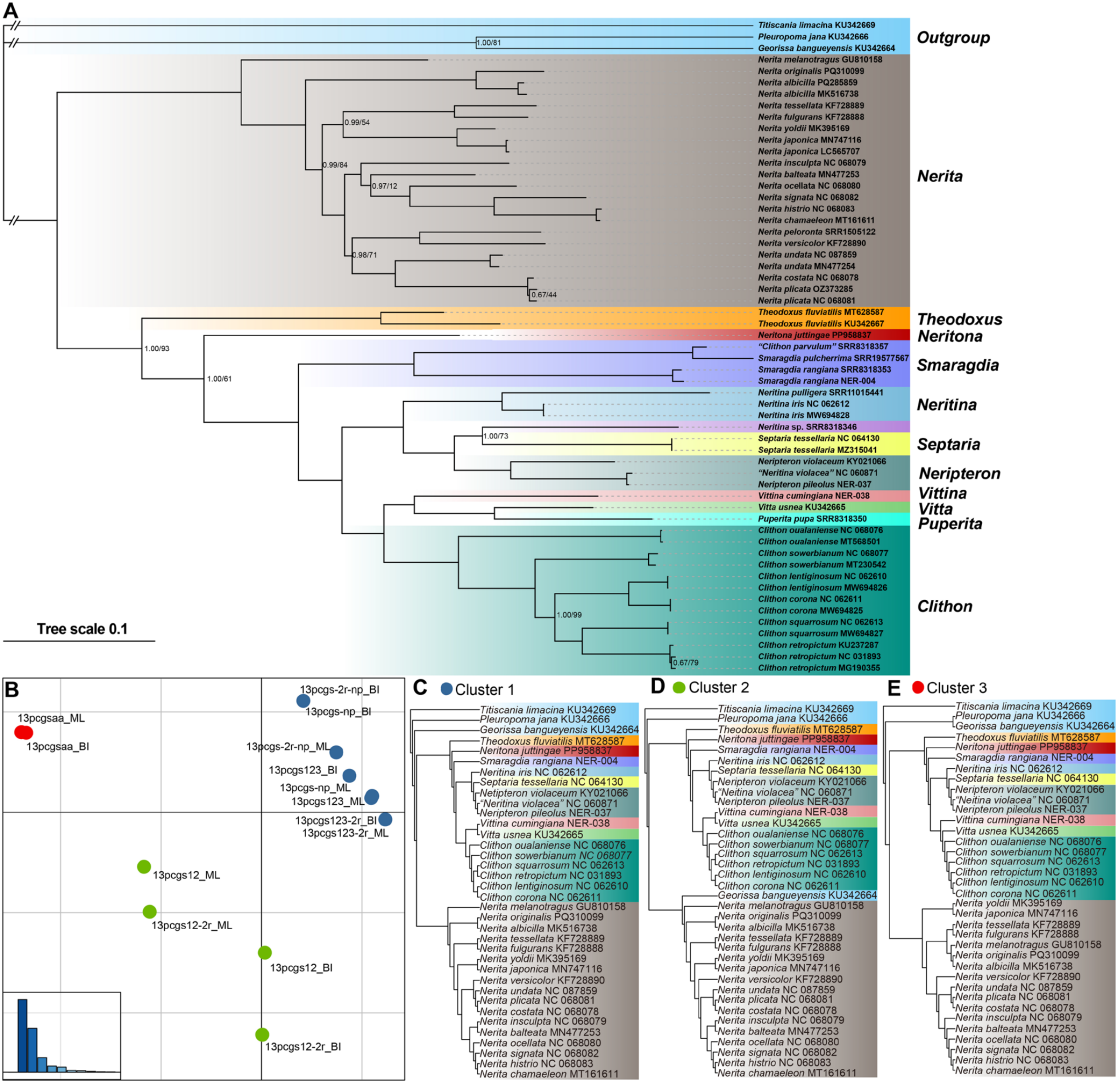
Maximum likelihood (ML) and Bayesian inference (BI) tree of Neritidae based on the “mitogenomes + RNA” dataset (A) and treespace analysis of phylogenies from seven “mitogenome” datasets (B–E). The GenBank accession numbers used are listed after the species names. The scale bar (0.1) corresponds to the estimated number of substitutions per site. Numbers at nodes are statistical support values for “BI posterior probabilities/ML bootstrap support”. “Unlabeled” denotes 100% bootstrap support. Color‐coded clades are different genera within Neritidae. (B) Two‐dimensional MDS plot of 10 trees, colored by different clusters. (C–E) For each cluster identified in B, a representative tree was selected.

The gene arrangement of the 13 PCGs and 2 rRNAs within the family Neritidae is completely consistent (Lin et al. 2026) and cannot provide useful phylogenetic information. A total of 10 ML and BI trees were inferred from five mitogenome datasets of Neritidae. These 10 trees were grouped into three clusters, representing three distinct tree types (see Figure 5B–E). As illustrated in Figure 5B, the choice of the dataset exerted a more substantial effect on the resulting tree topology than the choice of phylogenetic inference method. The most common topology, Cluster 1, is shown in Figure 5C. An extra phylogenetic tree including more mitogenome sequences and mitogenome sequences assembled from transcriptome data was chosen as the main figure (Figure 5A). The structure of three clusters is generally consistent, except the position of outgroup sequence *Georissa bangueyensis* (KU342664) is different in Cluster 2, and *Nerita melanotragus* (GU810158) is different in Cluster 3.

The phylogenetic tree included representatives from 12 genera of Neritidae (Figure 5A) and recovered two primary clades. Clade A comprised the marine genus *Nerita*, whereas Clade B contained the remaining sampled genera (*Theodoxus*, *Neritona*, *Smaragdia*, *Neritina*, *Septaria*, *Neripteron*, *Vittina*, *Vitta*, *Puperita*, *Clithon*, and one undetermined lineage). All three newly assembled mitogenomes fell within Clade B. *Smaragdia rangiana* (NER‐004) clustered with another *S*. *rangiana* mitogenome assembled from transcriptome data (SRR8318353) and with an additional *Smaragdia* sequence; a transcriptome‐derived sequence labeled as “*Clithon parvulum*” (SRR8318357) was placed within this clade. *Neripteron pileolus* (NER‐037) clustered with a record labeled as “*Neritina violacea*” (NC_060871) and with *Neripteron violaceum* (KY021066). *Vittina cumingiana* (NER‐038) formed a distinct lineage sister to *Vitta* and *Puperita*.

## 4 Discussion

Our phylogenetic analyses support the division of Neritidae into two subfamilies, Neritinae and Neritininae, consistent with previous mitogenome‐based studies (Feng et al. 2021; Miao et al. 2022; Qi et al. 2023; Lin et al. 2026). Clade A (Figure 5) contained only the marine genus *Nerita* and corresponds to Neritinae. Clade B comprised genera of Neritininae, most of which inhabit freshwater or brackish environments and often show amphidromous life histories (Ford 1979; Eichhorst 2016a, 2016b).

Phylogenetic relationships were generally stable across datasets and inference methods, with a few exceptions (e.g., the placement of *Nerita melanotragus* [GU810158] and the outgroup *Georissa bangueyensis* [KU342664]). Mitochondrial gene order was highly conserved across Neritidae and therefore provided little additional phylogenetic signal. This conservation, together with limited taxon sampling for several genera, suggests that mitogenomes alone may be insufficient to fully resolve relationships within the family. Moreover, current trees remain inadequate for testing links between habitat shifts and diversification in Neritidae. The three species involved in this study generally share similar habitats with their congeners: *S. rangiana* is marine, *N. pileolous* is brackish, and *V. cumingiana* is freshwater and brackish. Notably, *Smaragdia* is the only completely marine genus in the Neritininae clade, which may suggest a secondary transition from freshwater/brackish to marine habitat. However, this transformation may not have occurred only a few times. Holthuis (1995) suggested at least 12 transitions among marine, brackish, and freshwater habitats within Cycloneritida, and Qi et al. (2023) also inferred repeated habitat transitions. Broader taxon sampling and integration of nuclear genomic data will be required to robustly reconstruct the evolutionary history of all 16 neritid genera.

All three newly assembled mitogenomes belonged to Clade B. *Smaragdia rangiana* (NER‐004) clustered with other *Smaragdia* sequences and with a transcriptome‐derived record labeled as “*Clithon parvulum*” (SRR8318357), whereas other *Clithon* species formed a separate monophyletic group. This pattern suggests that SRR8318357 may be mislabeled. Similarly, *Neripteron pileolus* (NER‐037) clustered most closely with a record labeled as “*Neritina violacea*” (NC_060871, a junior synonym of *Neripteron violaceum*) rather than with the authenticated *N*. *violaceum* sequence (KY021066), again suggesting potential misidentification or the use of historical synonyms. *Vittina cumingiana* (NER‐038) represents the first available mitogenome for *Vittina*; additional sampling will be needed to test the monophyly and internal relationships of this genus.

In our phylogenetic analyses, the outgroup *Georissa bangueyensis* (Hydrocenidae) showed unstable placement across different datasets, clustering inconsistently in TreeSpace analysis (Cluster 2, Figure 5B). This instability, together with the considerable phylogenetic distance between Hydrocenidae and Neritidae, raises the possibility of long‐branch attraction (LBA) artifacts (Bergsten 2005). Ideally, a more closely related outgroup within Neritimorpha (e.g., Phenacolepadidae or a broader representation of Neritopsidae) would be used to root the tree. However, complete mitogenomes for these families are currently unavailable in public databases. We encourage future studies to include additional neritimorph mitogenomes to mitigate potential LBA effects.

Voucher metadata for “*Clithon parvulum*” (SRR8318357; voucher MCZ:Mala:386153) indicate collection from seagrass blades (Harvard University and Morris 2026), a typical habitat of *Smaragdia*, whereas *Clithon parvulum* is generally reported from freshwater streams. Notably, *Smaragdia paulucciana* (Gassies, 1870) is a junior synonym of *Clithon parvulum* (Le Guillou, 1841) (Eichhorst 2016b; MolluscaBase eds. 2026). Eichhorst (2016b) further noted that the name *S*. *paulucciana* has been inconsistently applied and in some cases refers to a distinct species, *Smaragdia patburkeae* Eichhorst, 2016. Together, these observations suggest that MCZ:Mala:386153 may represent a misidentified specimen of *Smaragdia*. Unfortunately, the voucher record lacks photographs, and the shell was broken for soft tissue extraction, preventing re‐examination. A similar problem may occur in the *Neripteron* clade. *Neripteron violaceum* (often listed under its synonym *Neritina violacea*) and *N*. *pileolus* have been confused in the literature. For example, Tchang et al. (1964) described *Neritina violacea* with traits consistent with *N*. *pileolus* (yellow‐brown shell, bluish‐gray aperture and parietal shelf, and irregular zigzag patterns), whereas Li (2019) illustrated a typical *N*. *violaceum* specimen under the name *N*. *pileolus*. Because the NCBI record NC_060871 lacks locality, voucher information, and images, we can only infer its identity indirectly from molecular placement. Ideally, definitive confirmation of these cases would require re‐examination of the original vouchers. However, the voucher for SRR8318357 (MCZ:Mala:386153) was dissected and is no longer intact (Harvard University and Morris 2026), and NC_060871 lacks any voucher information in its GenBank record. Therefore, we present the classification corrections suggested in this paper only as provisional recommendations, warranting future testing with newly collected, well‐vouchered specimens accompanied by accessible images and locality data. For other species labeled as their synonyms in the database mentioned by Lin et al. (2026), we verified them using MolluscaBase (MolluscaBase eds. 2026).

After excluding apparently mislabeled records from *Smaragdia*, *Neripteron*, and *Neritina*, the genera sampled in this study were recovered as monophyletic. This emphasizes that careful verification of species identifications is essential for mitogenome‐based phylogenetics. We recommend that future submissions to public databases include voucher information, clear specimen images, and key diagnostic characters to support downstream comparative and phylogenetic analyses.

## Author Contributions

**Yiyong Rao:** data curation (equal), funding acquisition (lead), visualization (equal), writing – original draft (equal), writing – review and editing (equal). **Yuanzheng Meng:** methodology (equal), writing – original draft (equal), writing – review and editing (equal). **Sheng Zeng:** formal analysis (equal), visualization (equal), writing – original draft (equal), writing – review and editing (equal). **Deyuan Yang:** conceptualization (lead), data curation (equal), formal analysis (equal), investigation (lead), methodology (equal), project administration (lead), supervision (lead), writing – original draft (equal), writing – review and editing (equal).

## Funding

This work was supported by the National Natural Science Foundation of China (42206119) and the Central Public‐Interest Scientific Institution Basal Research Fund, South China Sea Fisheries Research Institute, CAFS (2024RC08).

## Conflicts of Interest

The authors declare no conflicts of interest.

## Supporting information


**Table S1:** Original and TrimAl lengths of 13 PCGs, 2 rRNAs, and 13 PCGsAA sequences.
**Table S2:** Best partitioning schemes and models based on different datasets for maximum likelihood and Bayesian inference analysis.
**Table S3:** The Blast results of mtgenome, COX1 and 16S (Top 10 blast results are shown).
**Table S4:** Codon numbers and relative synonymous codon usage (RSCU) of 13 PCGs in three mitogenomes.

**Data S2:** Preliminary Maximum Likelihood (ML) and Bayesian Inference (BI) phylogenetic trees of Neritidae based on the 13PCGs + 2rRNA dataset.

## Data Availability

The data generated in this study have been deposited as follows: the complete mitochondrial genomes in NCBI GenBank under the accession numbers PX869896 for *Smaragdia rangiana*, PX869897 for *Neripteron pileolus*, and PX869898 for *Vittina cumingiana*.
