# Supplementary figures and images for: Three New Mitochondrial Genomes of Neritidae (Gastropoda, Neritimorpha) From China and Insights Into Their Phylogenetic Position
Source: Ecol Evol. 2026 Jul 2;16(7):e73888. doi: 10.1002/ece3.73888 (PMC13327798; doi:10.1002/ece3.73888)

ML

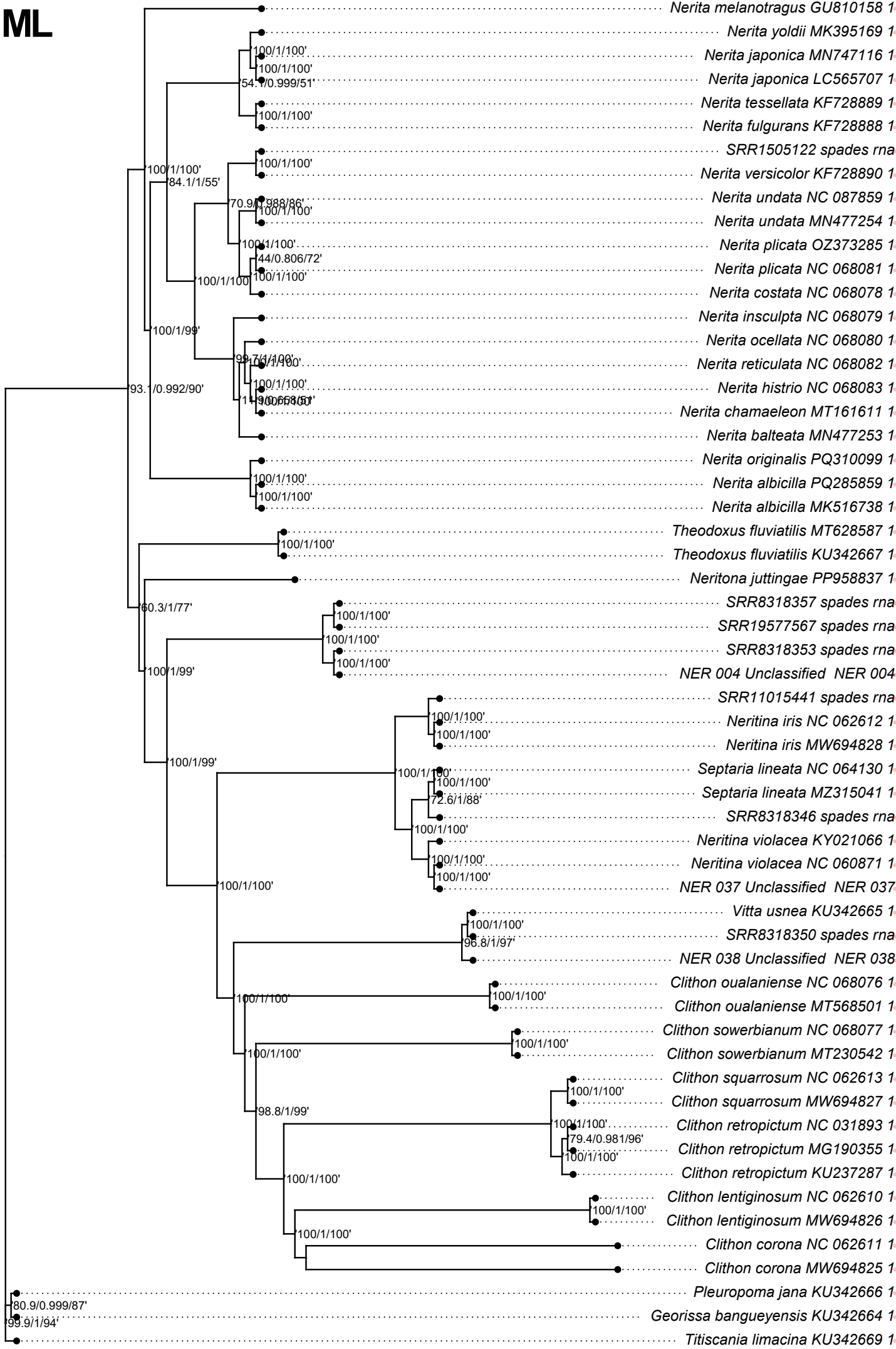

BI

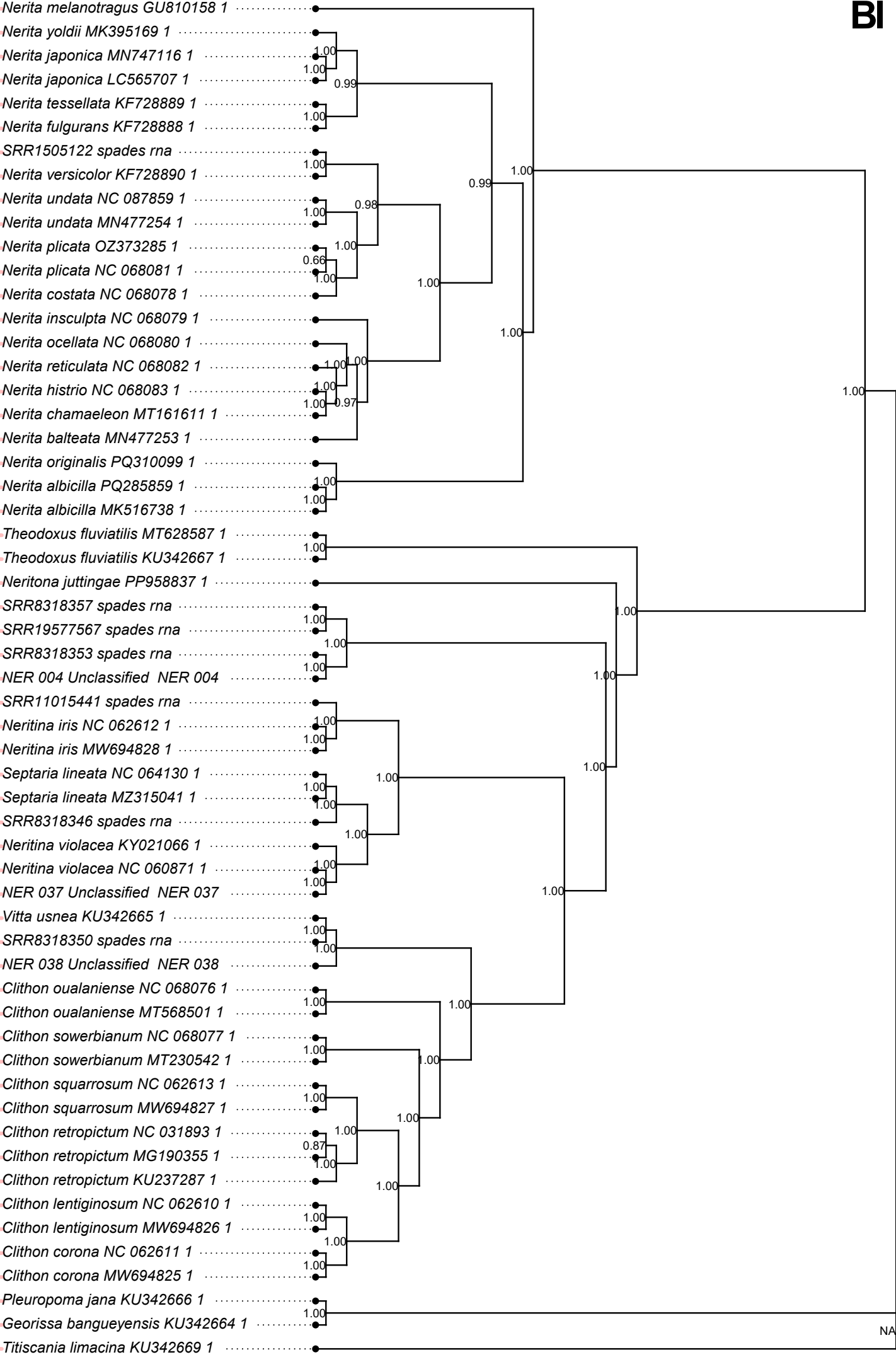

NA

Supplement: Supplementary file 2 — Data S2: Preliminary Maximum Likelihood (ML) and Bayesian Inference (BI) phylogenetic trees of Neritidae based on the 13PCGs + 2rRNA dataset. [file ECE3-16-e73888-s002.pdf]
